# The association between network literacy and subjective well-being among middle-aged and older adults

**DOI:** 10.3389/fpsyg.2025.1590622

**Published:** 2025-05-29

**Authors:** Lin Sun, Juan Xiong, Chong Zhang

**Affiliations:** ^1^Research Institute of Social Development, Southwestern University of Finance and Economics, Chengdu, Sichuan, China; ^2^Institute of Network Social Governance, School of Marxism, University of Electronic Science and Technology of China, Chengdu, Sichuan, China

**Keywords:** network literacy, middle-aged and older adults, subjective well-being, China Family Panel Studies (CFPS), interpersonal relationships

## Abstract

**Introduction:**

With the advent of the digital age, network literacy has become a pivotal indicator for assessing the social adaptability and quality of life among middle-aged and older adults.

**Methods:**

This study is based on data from the Chinese Family Panel Studies (CFPS), utilizing three waves of data from 2014, 2018, and 2020. We employ a fixed-effects panel regression and Bootstrap method to explore the association between network literacy and subjective well-being of this demographic and its mediating mechanisms.

**Results:**

The research findings indicate a significant positive correlation between network literacy and subjective well-being among middle-aged and older adults, with both network usage literacy and network perception literacy positively promoting their subjective well-being. Interpersonal relationships play a positive mediating role between network literacy and subjective well-being. Furthermore, the frequency of online social interaction and entertainment in network usage literacy, as well as the importance of these activities in network perception literacy, have significant positive relationships with subjective well-being.

**Conclusion:**

These findings underscore the importance of enhancing network literacy among middle-aged and older adults, providing a foundation for policymakers and community organizations to facilitate their integration into the digital society. This integration can enable them to enjoy the Internet's convenience and entertainment, thereby improving their quality of life and well-being. Future research should extend its focus to the multidimensional aspects of network literacy and its enduring effects on the well-being.

## 1 Introduction

The rapid development of information technology has transformed the Internet into a vital platform for accessing information, communication, learning, and entertainment. As the digital age progresses, the ability to effectively navigate and utilize the Internet has become increasingly important for individuals of all ages. This ability, known as network literacy, is defined as the capacity to effectively use and critically evaluate information found online, including skills such as information filtering, privacy protection, and network security (Miles, 2007; Hopkins, 2022; Tinmaz et al., 2022). For middle-aged and older adults, improving network literacy can not only enhance their ability to utilize online resources but also meet their needs in various aspects such as learning, working, socializing, entertaining, and business, thereby improving their quality of life and well-being.

According to the 55th statistical report on China's Internet development released by the China Internet Network Information Center (CNNIC), as of January 2025, the number of Chinese netizens has reached 1.108 billion people, among which the number of netizens aged 50 and above is as high as 378 million people, accounting for 34.1% of the total netizen population [China Internet Network Information Center (CNNIC), 2025]. These data indicate that middle-aged and older adults have become a significant component of China's internet user population. However, constrained by various factors such as age, educational background, and lifestyle habits (Klimova et al., 2021), this demographic generally exhibits a deficiency in network literacy. They encounter a range of challenges in their Internet usage, including inadequate information acquisition skills, a lack of proficiency in online operations, and a weak sense of Internet security (Friemel, 2016). These challenges restrict middle-aged and older adults from enjoying the convenience of the Internet and may also weaken their social interactions and participation, thereby affecting their subjective well-being. Improving network literacy can enhance their ability to obtain health, entertainment, and social information, enriching their daily lives. For example, through online learning platforms, they can acquire new knowledge and skills to boost their sense of self-efficacy. Watching short videos and engaging in online entertainment activities can bring them pleasure and improve their psychological state (Zhang R. et al., 2024). Meanwhile, enhanced network literacy helps them use social platforms to stay connected with family and friends, reducing loneliness and strengthening social support (Zhang C. et al., 2022). This increased social engagement not only improves their emotional state but also enhances their sense of social identity and belonging, thus significantly boosting their subjective well-being. Therefore, enhancing the network literacy of middle-aged and older adults and helping them overcome these difficulties is of great significance for strengthening their social adaptability, improving the quality of life, and ultimately enhancing their overall well-being.

A review of the literature reveals that there are few studies that directly examine the impact of network literacy on the well-being of middle-aged and older adults, most focus on the perspectives of digital literacy or Internet use. In terms of digital literacy, a quasi-experimental study conducted among older adults in South Korea found that there is a correlation between improved digital literacy and the enhancement of well-being (Lee et al., 2022). Similarly, another experimental study among older adults in Chile reached the same conclusion (Carrasco-Dajer et al., 2024). However, a non-randomized controlled study among older adults in Singapore found that an increase in personal digital literacy did not lead to an improvement in well-being (Ngiam et al., 2022). In terms of Internet use, although many scholars have discussed its impact on the well-being of middle-aged and older adults in recent years, the conclusions remain controversial (Zhang C. et al., 2024). Some studies indicate a significant positive effect. For instance, a quasi-experimental study conducted in Israel revealed that Internet use can enhance the well-being of older adults by shaping their interpersonal interactions (Shapira et al., 2007). Similarly, an analysis of longitudinal data from the New Zealand Health, Work, and Retirement Study (NZHWR) suggested that Internet use contributes to improved well-being in older adults by fostering social engagement and mitigating feelings of loneliness (Szabo et al., 2019). Some Studies utilizing Chinese data have also identified a substantial positive correlation between Internet usage and the well-being of middle-aged and older adults (Lu and Kandilov, 2021; Li and Zhou, 2021; Ye et al., 2022; Jin et al., 2023). However, these findings are not universally consistent. A randomized, controlled intervention study had reported no discernible positive or negative influence of Internet use on the well-being of older adults (Slegers et al., 2008). Echoing this, a cross-sectional study utilizing data from the National Health and Aging Trends Study (NHATS) found no association between Internet use and the well-being of older adults (Elliot et al., 2014). Furthermore, research based on the GErontological Regional DAtabase and Resource Center (GERDA) project indicated that, after accounting for control variables, Internet use did not exhibit a statistically significant correlation with well-being (Viklund and Forsman, 2022). An examination of three waves of data (2013, 2016, and 2019) from the Japan Gerontological Evaluation Study (JAGES) also yielded little or no strong evidence linking Internet use to well-being indicators (Nakagomi et al., 2022). Additionally, the pervasive engagement with the Internet could potentially diminish face-to-face interactions (Kraut et al., 1998), exacerbate feelings of solitude (Nie, 2001), and consequently, lower the older adults' life satisfaction and well-being (Yang et al., 2021).

In summary, previous research has laid a solid theoretical foundation and empirical experience for this study, providing a scientific basis for the selection of control variables. Nevertheless, we have identified certain deficiencies in previous studies: (1) There is a scarcity of research examining the nexus between network literacy and well-being, predominantly focusing on Internet use and employing simplistic measurement approaches. (2) The different impacts of the specific types of Internet use on the well-being of middle-aged and older adults also deserve further discussion. (3) Most prior studies have relied on regional survey or cross-sectional data, and causal relationships are worth discussing. Based on this, this study aims to use extensive longitudinal survey data to explore in depth the relationship between online literacy and subjective well-being of middle-aged and older adults. We comprehensively measure network literacy and provide detailed arguments based on various dimensions and indicators.

## 2 Methods

### 2.1 Data sources

The data for this study are sourced from the China Family Panel Studies (CFPS), which aims to track and collect data at the individual, family, and community levels to reflect changes in Chinese society, economy, population, education, and health, providing a data foundation for academic research and public policy analysis. The CFPS sample covers 25 provinces/cities/autonomous regions in Chinese Mainland, with a target sample size of 16,000 households, and the respondents include all family members in the sample households. Considering the absence of some important variables, we ultimately select samples from 2014, 2018, and 2020. After removing missing values and singular values, the total sample size of middle-aged and older adults aged 45 and above is 42,103, with 15,355, 15,921, and 10,827 in 2014, 2018, and 2020, respectively.

### 2.2 Variables

#### 2.2.1 Dependent variable

The dependent variable is residents' subjective well-being. Respondents were asked to rate their happiness on a scale from 0 to 10 according to the CFPS questionnaire, where 0 represents the lowest level and 10 represents the highest level of subjective well-being.

#### 2.2.2 Independent variable

The independent variable of this article is network literacy. The criteria for selecting indicators of network literacy are based on the CFPS questionnaire, which covers a comprehensive range of internet-related activities and perceptions that reflect the actual usage and attitudes of middle-aged and older adults. The specific indicators include the frequency of online activities such as learning, working, social interaction, entertainment, and business activities, as well as the perceived importance of these activities in daily life. Therefore, it is divided into two dimensions: network usage literacy and network perception literacy, with specific indicators detailed in Table 1. The measurement of network literacy in 2020 has some differences compared to 2014 and 2018, but overall it can still reflect the level of network literacy among middle-aged and older adults. The Cronbach's alpha coefficients for the reliability analysis of network literacy in 2014, 2018, and 2020 are 0.938, 0.935, and 0.903, respectively; The KMO values for validity analysis are 0.890 (*P* < 0.001), 0.883 (*P* < 0.001), and 0.954 (*P* < 0.001), respectively. All test values exceed 0.7, indicating good reliability and validity of network literacy. Both network usage literacy and network perception literacy were weighted using Entropy-TOPSIS method, which calculates the weight coefficients and scores separately for each year to account for potential changes in the importance of different indicators over time. This method objectively assigns weights based on the information content of each indicator, avoiding subjectivity and ensuring a comprehensive evaluation. The scores for each indicator are normalized and combined to obtain a comprehensive network literacy score for each individual in each year. The differences in scores for each indicator between 2020, 2014, and 2018 are handled by this normalization process, which ensures comparability across different years. The obtained scores are used to measure the level of network literacy of middle-aged and older adults. Scores range from 0 to 1, with higher values indicating higher levels of network literacy.

**Table 1:** The measured items of network literacy.

**Primary indicators**	**2014, 2018**		**2020**	
	**Sub-indicators**	**Value**	**Sub-indicators**	**Value**
Network usage literacy	Whether to use the Internet	0–1	Whether to use the Internet	0–1
	Frequency of online learning	0–6	Daily Internet usage duration (hours)	0–16.5
	Frequency of online work	0–6	Whether to play online games	0–1
	Frequency of online social interaction	0–6	Whether to shop online	0–1
	Frequency of online entertainment	0–6	Whether to watch short videos	0–1
	Frequency of online business activities	0–6	Whether to learn online	0–1
	Frequency of logging into email	0–7	Whether to use WeChat	0–1
Network perception literacy	Importance of online learning	0–5	Importance of online learning	0–5
	Importance of online work	0–5	Importance of online work	0–5
	Importance of online social interaction	0–5	Importance of online social interaction	0–5
	Importance of online entertainment	0–5	Importance of online entertainment	0–5
	Importance of online business activities	0–5	Importance of online in daily Life	0–5

#### 2.2.3 Mediating variable

In this study, we select interpersonal relationships as the mediating variable to explore the relationship between network literacy and subjective well-being. Previous research has shown that interpersonal relationships play a crucial role in mediating the impact of various factors on subjective well-being. For example, a study by Zheng and Shen (2025) explores the association between social comparison and subjective well-being, highlighting the mediating role of interpersonal relationships in this context. Some previous literature has also found that Internet use can affect well-being by expanding social interaction among older adults (Cotten et al., 2013; Yu et al., 2021). These studies suggest that individuals with higher network literacy are more likely to engage in positive social interactions, which in turn enhances their subjective well-being. Therefore, this study chooses interpersonal relationships as the mediating variable, and based on the CFPS questionnaire's “How good are your interpersonal relationships?”, respondents score it, with 0 representing the worst interpersonal relationships and 10 representing the best interpersonal relationships.

#### 2.2.4 Covariates

Combining previous studies, five categories of control variables are selected: personal characteristics, lifestyle habits characteristics, socioeconomic characteristics, family characteristics, and psychological characteristics. Personal characteristics include the following variables: gender, age, place of residence, educational attainment, marital status, and chronic diseases. Gender, place of residence, marital status, and chronic diseases are all treated as binary variables, assigned a value of 1 for males and 0 for females. Place of residence is assigned a value of 1 for urban areas and 0 for rural areas. If there is a spouse, it is 1; if there is no spouse, it is 0. Having chronic diseases is 1, and having no chronic diseases is 0. Age is a continuous variable, ranging from 45 to 102 years old. Educational attainment is categorized into five levels: illiteracy, primary education, junior secondary education, senior secondary education, and university or higher. These levels are assigned numerical values of 1, 2, 3, 4, and 5, respectively. Lifestyle habits characteristics include the following variables: drinking, smoking, and late sleep behavior, all of which are processed as binary variables. Drinking more than three times a week in the past month is assigned as 1, and never drinking or drinking no more than three times a month is assigned as 0. smoking in the past month is assigned as 1 and not smoking is assigned as 0. Going to bed later than 11 p.m. is defined as late sleep behavior, assigned a value of 1, otherwise assigned a value of 0. Socioeconomic characteristics: self-assessed social status and self-assessed income level, both rated on a scale of 1–5, where 1 represents the lowest and 5 represents the highest. Family characteristics: the number of family members, ranging from 1 to 21. Psychological characteristics: psychological expectations, expressed as “the degree of confidence in one's own future”, and the score is also from 1 to 5, where 1 represents the lowest confidence and 5 represents the highest.

### 2.3 Statistical analysis methods

SPSS 27 and Stata 15.0 software are used to conduct statistical analysis of the research data. Specifically, it is divided into three steps: The first step is to determine the fixed-effects panel regression results based on the Hausman test method, the Hausman test results for the impact of network usage literacy on subjective well-being show a chi-square statistic of 517.70, with a *p*-value of 0.0000. The Hausman test results for the impact of network perception literacy on subjective well-being show a chi-square statistic of 524.46, with a *p*-value of 0.0000. Subsequently, robustness checks and heterogeneity analysis are conducted. The second step is to use the Bootstrap method to test and estimate the mediating effect of interpersonal relationships between network literacy and the well-being of middle-aged and older adults. The third step is to explore the impact of specific indicators under the two dimensions of network literacy on their well-being.

## 3 Results

### 3.1 Descriptive statistics

Table 2 shows the descriptive statistics of all variables. From 2014 to 2020, both the subjective well-being and network literacy among middle-aged and older adults improved, especially the network perception literacy has improved significantly. Self-assessed social status, self-assessed income level, and psychological expectations have also improved. The proportion of middle-aged and older adults who drank alcohol or smoked decreased. The average size of families has decreased. Other variables are relatively stable.

**Table 2:** Descriptive statistics.

**Variables**	**2014**		**2018**		**2020**	
	**Mean**	**S.D**	**Mean**	**S.D**	**Mean**	**S.D**
Subjective well-being	7.439	2.243	7.487	2.253	7.493	2.197
Network usage literacy	0.030	0.114	0.095	0.178	0.139	0.198
Network perception literacy	0.026	0.106	0.109	0.215	0.301	0.374
Gender	0.507	0.500	0.503	0.500	0.510	0.500
Age	58.478	9.710	58.990	9.666	58.686	9.301
Place of residence	0.467	0.499	0.477	0.500	0.488	0.500
Educational attainment	2.352	1.123	2.260	1.144	2.395	1.170
Marital status	0.875	0.330	0.876	0.329	0.885	0.319
Chronic diseases	0.238	0.426	0.242	0.428	0.224	0.417
Drinking	0.178	0.382	0.177	0.382	0.157	0.363
Smoking	0.312	0.463	0.309	0.462	0.292	0.455
Late sleep behavior	0.173	0.378	0.193	0.394	0.180	0.384
Self-assessed social status	3.033	1.030	3.269	1.128	3.310	1.091
Self-assessed income level	2.519	1.035	2.969	1.137	3.046	1.121
The number of family members	4.228	1.954	3.919	1.990	3.927	1.983
Psychological expectations	3.930	1.079	4.100	1.022	4.174	0.969
Interpersonal relationships	7.300	1.882	7.234	2.064	7.229	1.988
*N*	15,355		15,921		10,827	

### 3.2 Relationship between network literacy and subjective well-being

Table 3 shows the impact of network literacy on subjective well-being of middle-aged and older adults under the fixed-effects panel regression. The results demonstrate a significant positive association between network literacy and subjective well-being. Both network usage literacy and network perception literacy have positive effects (β = 0.221, *P* < 0.05; β = 0.135, *P* < 0.05). It can be concluded that the higher the network literacy is, the stronger the well-being of middle-aged and older adults becomes. In addition, we evaluate the impact of control variables on subjective well-being. According to the regression results, middle-aged and older adults with spouses have stronger subjective well-being compared to those without spouses. At the same time, self-assessed social status and self-assessed income level also have significant positive impacts on subjective well-being. At the psychological level, psychological expectations also have a positive effect.

**Table 3:** Regression results of the impact of network literacy on subjective well-being (*N* = 42,103).

**Variables**	**(1)**	**(2)**	**(3)**	**(4)**
Network usage literacy	0.248 (0.115)^*^	0.221 (0.107)^*^		
Network perception literacy			0.199 (0.063)^**^	0.135 (0.059)^*^
Gender	0.236 (0.649)	0.378 (0.604)	0.244 (0.649)	0.382 (0.604)
Age	−0.010 (0.005)^*^	0.002 (0.005)	−0.014 (0.005)^**^	0.00005 (0.005)
Place of residence	−0.011 (0.077)	−0.005 (0.072)	−0.007 (0.077)	−0.003 (0.072)
Educational attainment	−0.014 (0.054)	−0.010 (0.050)	−0.022 (0.054)	−0.015 (0.050)
Marital status	0.344 (0.085)^***^	0.355 (0.079)^***^	0.344 (0.085)^***^	0.355 (0.079)^***^
Chronic diseases	−0.036 (0.033)	−0.020 (0.031)	−0.036 (0.033)	−0.020 (0.031)
Drinking	−0.006 (0.049)	0.032 (0.045)	−0.005 (0.049)	0.033 (0.045)
Smoking	0.137 (0.062)^*^	0.094 (0.058)	0.138 (0.062)^*^	0.095 (0.058)
Late sleep behavior	−0.071 (0.043)	−0.074 (0.040)	−0.069 (0.043)	−0.072 (0.040)
Self-assessed social status	0.147 (0.015)^***^	0.085 (0.014)^***^	0.147 (0.015)^***^	0.084 (0.014)^***^
Self-assessed income level	0.075 (0.014)^***^	0.074 (0.013)^***^	0.076 (0.014)^***^	0.075 (0.013)^***^
The number of family members	0.002 (0.007)	−0.001 (0.007)	0.002 (0.007)	−0.001 (0.007)
Psychological expectations	0.407 (0.015)^***^	0.332 (0.014)^***^	0.407 (0.015)^***^	0.332 (0.014)^***^
Interpersonal relationships		0.378 (0.007)^***^		0.378 (0.007)^***^
Constant	5.291 (0.494)^***^	2.280 (0.463)^***^	5.506 (0.503)^***^	2.397 (0.471)^***^
*R* ^2^	0.059	0.185	0.059	0.185
*Prob > F*	0.000	0.000	0.000	0.000

^*^p < 0.05.

^**^p < 0.01.

^***^p < 0.001.

Standard errors in parentheses.

### 3.3 Robustness check

In order to reduce endogeneity estimation bias caused by reverse causality as well as omitted variables, by synthesizing previous relevant studies (Lei et al., 2023; Ma et al., 2024; Zhou et al., 2024), the interaction term between the per capita number of fixed-line telephones in each province in 1984 and the per capita number of Internet broadband subscribers in the previous year is used as an instrumental variable for digital literacy for verification. The regression results shown in Table 4 indicate that the instrumental variables in the first-stage regression results significantly contribute to the level of middle-aged and elderly people's network usage literacy and network perception literacy at the 1‰ level, and the *F*-value is >10, indicating that there is no weak instrumental variable problem. Meanwhile, the second-stage regression results, compared with the benchmark regression results, the original conclusions remain unchanged after controlling for endogeneity, which verifies that the regression results in this paper are robust.

**Table 4:** Instrumental variable estimation results (*N* = 42,103).

**Variables**	**(5)**		**(6)**	
	**First stage**	**Second stage**	**First stage**	**Second stage**
Network usage literacy		1.109 (0.447)^*^		
Network perception literacy				0.632 (0.255)^*^
IV	0.212 (0.010)^***^		0.372 (0.015)^***^	
Control covariates	Yes	Yes	Yes	Yes
F value of the first stage	451.693		616.405	
*R* ^2^	0.254	0.304	0.198	0.305
*Prob > F*	0.000	0.000	0.000	0.000

^*^p < 0.05.

^***^p < 0.001.

Standard errors in parentheses.

Furthermore, in order to improve the reliability of research conclusions, the method of replacing dependent variables is adopted to test the robustness of the model and avoid bias caused by a single measurement of subjective well-being. Due to the strong negative correlation between depression and well-being (Chuning et al., 2024), it can indirectly reflect the level of well-being to a certain extent. In the past, some literature also used depression to measure the well-being of middle-aged and older adults (Lifshitz et al., 2018; Tkatch et al., 2021). Depression is assessed using the Entropy-TOPSIS method based on the simplified depression scale from the CFPS questionnaire, with final scores ranging from 0 to 1. A higher score indicates a greater level of depression. The regression results of network usage literacy and network perception literacy on depression among middle-aged and older adults are shown in Table 5. Both have significant negative impacts on depression, indicating that the higher the network literacy, the lower the level of depression, further verifying that the higher the network literacy, the stronger the well-being.

**Table 5:** Regression results of the impact of network literacy on depression (*N* = 42,103).

**Variables**	**(5)**	**(6)**
Network usage literacy	−0.028 (0.009)^**^	
Network perception literacy		−0.044 (0.005)^***^
Control covariates	Yes	Yes
*R* ^2^	0.165	0.168
*Prob > F*	0.000	0.000

^**^p < 0.01.

^***^p < 0.001.

Standard errors in parentheses.

### 3.4 Heterogeneity analysis

Considering the significant heterogeneous characteristics within the middle-aged and elderly group, the group differences in the impact of network literacy on well-being were examined in depth through group regression analysis. The results in Table 6 show that in the gender dimension, network literacy significantly enhances the subjective well-being of male middle-aged and elderly people, but the effect on the female group does not reach the level of statistical significance; in the urban-rural difference, network literacy significantly enhances the well-being of rural middle-aged and elderly people, but the well-being brought about by network literacy is not significant for urban middle-aged and older adults; in the aspect of the education level, the network usage literacy and the network perception literacy are only significantly positively correlated in the group with education level below high school, but not significant in the group with education level of high school and above. Self-assessed income stratification analysis reveals that network literacy has a general enhancement effect on the well-being of the low-income group, while it shows a differential effect in the high-income group, with only network perception literacy showing a significant effect. These findings provide an important basis for formulating precise digital literacy enhancement policies.

**Table 6:** Heterogeneity analysis (*N* = 42,103).

**Variables**	**Gender**		**Urban**		**Educational attainment**		**Self-assessed income level**	
	**Males**	**Females**	**Urban areas**	**Rural areas**	**Below high school**	**High school or above**	**Low-income**	**High-income**
Network usage literacy	0.230 (0.078)^**^	0.035 (0.098)	0.037 (0.079)	0.428 (0.111)^***^	0.374 (0.081)^***^	0.035 (0.083)	0.186 (0.069)^**^	0.151 (0.138)
Control covariates	Yes	Yes	Yes	Yes	Yes	Yes	Yes	Yes
*R* ^2^	0.324	0.295	0.202	0.2942	0.299	0.370	0.300	0.260
*Prob > F*	0.000	0.000	0.000	0.000	0.000	0.000	0.000	0.000
Network perception literacy	0.173 (0.049)^***^	0.042 (0.059)	0.059 (0.052)	0.242 (0.061)^***^	0.219 (0.046)^***^	0.023 (0.060)	0.124 (0.043)^**^	0.162 (0.080)^*^
Control covariates	Yes	Yes	Yes	Yes	Yes	Yes	Yes	Yes
*R* ^2^	0.324	0.295	0.202	0.2942	0.299	0.370	0.300	0.260
*Prob > F*	0.000	0.000	0.000	0.000	0.000	0.000	0.000	0.000
*N*	21,321	20,782	20,057	22,046	35,305	6,798	32,999	9,104

^*^p < 0.05.

^**^p < 0.01.

^***^p < 0.001.

Standard errors in parentheses.

### 3.5 Mediating effect analysis

The benchmark regression results reveal that interpersonal relationships have a positive impact on subjective well-being. Therefore, the bootstrap method is further used to investigate the mediating effect of interpersonal relationships on the relationship between network literacy and subjective well-being among middle-aged and older adults. The results are shown in Table 7. The mediating effect analysis shows that the total effects of network usage literacy and network perception literacy on subjective well-being are 0.405 and 0.413, respectively. Among them, the indirect effects through interpersonal relationships are 0.108 and 0.137, while the direct effects are 0.297 and 0.276, respectively. This means that interpersonal relationships to some extent mediate the impact of network literacy on subjective well-being, with indirect effects accounting for 26.643 and 31.612% of the total effects. Therefore, these results indicate that interpersonal relationships play an important mediating role between network literacy and subjective well-being.

**Table 7:** Mediating effect of interpersonal relationships on the relationship between network literacy and subjective well-being (*N* = 42,103).

**Mediating effect**	**Network usage literacy**			**Network perception literacy**		
	**Observed coef**.	**Std. err**.	**[95% conf. interval]**	**Observed coef**.	**Std. err**.	**[95% conf. interval]**
Total	0.405	0.060	[0.287, 0.523]	0.413	0.042	[0.331, 0.494]
Indirect	0.108	0.023	[0.062, 0.154]	0.137	0.016	[0.105, 0.168]
Direct	0.297	0.056	[0.188, 0.406]	0.276	0.038	[0.201, 0.351]
Proportion of mediating effect	26.643%			31.612%		

### 3.6 Relationship between sub-indicators of network literacy and subjective well-being

Due to the differences between middle-aged and older adults in their own quality, hobbies and other aspects, different Internet use purposes and Internet perception will have different impacts on their well-being. Given this, it is necessary to further explore the impact of sub-indicators of network usage literacy and network perception literacy on subjective well-being. These sub-indicators are sourced from Table 1. Considering the comparability of network usage literacy across 2014, 2018, and 2020, six common sub-indicators are selected for 2014 and 2018: frequency of online learning, frequency of online work, frequency of online social interaction, frequency of online entertainment, frequency of online business activities, frequency of logging into email. For 2020, due to inconsistencies in the measurement questions, five representative sub-indicators are chosen: playing online games, shopping online, watching short videos, learning online, using WeChat. This study uses cross-sectional data to explore the relationships between these sub-indicators of network usage literacy and subjective well-being of middle-aged and older adults. For network perception literacy, four common sub-indicators across the 3 years were selected: importance of online learning, importance of online work, importance of online social interaction, importance of online entertainment, and we further use a fixed-effects panel regression to explore the relationships between these sub-indicators and subjective well-being. This can help us examine the different effects of different usage purposes and perceptions on subjective well-being.

Table 8 presents the estimated results of the relationship between sub-indicators of network usage literacy and subjective well-being. The results show that in 2014, there is a significant positive relationship between the frequency of online social interaction and subjective well-being among middle-aged and older adults (β = 0.033, *P* < 0.05). Frequency of online entertainment also demonstrates a significant positive association with subjective well-being (β = 0.037, *P* < 0.05). In 2018, Frequency of online social interaction and entertainment also have significant positive relationships with subjective well-being (β = 0.023, *P* < 0.001; β = 0.028, *P* < 0.001). In 2020, using the Internet to watch short videos has a significantly positive relationship with subjective well-being (β = 0.143, *P* < 0.001), using WeChat is also significantly positively correlated with subjective well-being (β = 0.182, *P* < 0.001). Watching short videos is an important way of online entertainment, and using WeChat is an important tool for online social interaction. These two activities not only enrich the daily lives of middle-aged and older adults, but also promote their social participation and emotional communication. Especially WeChat, as a bridge connecting family and friends, provides a convenient and fast communication platform, allowing middle-aged and older adults to share their daily life in a timely manner, strengthening their connections and emotional bonds with each other (Zhang and Liang, 2023). However, although the Internet provides rich resources and facilities for middle-aged and older adults, not all types of online activities can significantly improve their well-being. The study finds that the frequency of online learning, work, business activities, and logging into Email all have no significant relationships with subjective well-being. Playing online games, shopping online, and learning online also have no significant relationships.

**Table 8:** Relationship between sub-indicators of network usage literacy and subjective well-being.

**Sub-indicators**	**2014**	**2018**	**2020**	**Control covariates**	***R* ^2^**	***N***
Frequency of online learning	0.019 (0.016)			Yes	0.285	15,355
Frequency of online work	0.033 (0.018)			Yes	0.285	15,355
Frequency of online social interaction	0.033 (0.016)^*^			Yes	0.285	15,355
Frequency of online entertainment	0.037 (0.015)^*^			Yes	0.285	15,355
Frequency of online business activities	0.035 (0.040)			Yes	0.285	15,355
Frequency of logging into email	0.040 (0.026)			Yes	0.285	15,355
Frequency of online learning		0.003 (0.011)		Yes	0.322	15,921
Frequency of online work		0.018 (0.012)		Yes	0.322	15,921
Frequency of online social interaction		0.023 (0.007)^***^		Yes	0.322	15,921
Frequency of online entertainment		0.028 (0.008)^***^		Yes	0.322	15,921
Frequency of online business activities		0.001 (0.014)		Yes	0.322	15,921
Frequency of logging into email		−0.033 (0.024)		Yes	0.322	15,921
Playing online games			0.093 (0.080)	Yes	0.328	10,827
Shopping online			0.040 (0.052)	Yes	0.328	10,827
Watching short videos			0.143 (0.042)^***^	Yes	0.328	10,827
Learning online			0.038 (0.075)	Yes	0.328	10,827
Using WeChat			0.182 (0.042)^***^	Yes	0.329	10,827

^*^p < 0.05.

^***^p < 0.001.

Standard errors in parentheses.

Table 9 shows the estimated results of the relationship between sub-indicators of network perception literacy and subjective well-being. The fixed-effects panel regression results show that the importance of online social interaction has a significant positive impact on subjective well-being of middle-aged and older adults (β = 0.016, *P* < 0.01). The importance of online entertainment also has a significant positive impact (β = 0.025, *P* < 0.001).

**Table 9:** Relationship between sub-indicators of network perception literacy and subjective well-being (*N* = 42,103).

**Sub-indicators**	**(7)**	**(8)**	**(9)**	**(10)**
Importance of online learning	0.009 (0.007)			
Importance of online work		0.014 (0.007)		
Importance of online social interaction			0.016 (0.006)^**^	
Importance of online entertainment				0.025 (0.007)^***^
Control covariates	Yes	Yes	Yes	Yes
*R* ^2^	0.180	0.180	0.180	0.180

^**^p < 0.01.

^***^p < 0.001.

Standard errors in parentheses.

## 4 Discussion

This article uses longitudinal data from CFPS in 2014, 2018, and 2020 to explore the association between network literacy and subjective well-being of middle-aged and older adults. The research results show a significant correlation between network literacy and subjective well-being. Interpersonal relationships play a positive mediating role in the relationship between network literacy and subjective well-being. The classification regression of sub-indicators of network literacy has also obtained some detailed conclusions.

First of all, this study measures the series of questions about Internet use and perception as two dimensions of network literacy: network usage literacy and network perception literacy. This differs from previous studies that only focus on Internet use, and it is also different from the measurement of digital literacy. Network literacy focuses on the use level, skills and perception of the Internet, while digital literacy has a relatively wide range, including not only the Internet, but also other digital devices and tools, such as smart phones, tablets, digital media, etc. (Pegrum, 2010). Through fixed-effects panel regression analysis, it is found that both network usage literacy and network perception literacy have significant positive impacts on subjective well-being among middle-aged and older adults. That is, the stronger the network usage literacy and network perception literacy abilities of middle-aged and older adults, the stronger their subjective well-being. This finding provides a new perspective in contrast to traditional research. Previous studies have often focused solely on whether middle-aged and older adults use the internet and the frequency of their use, neglecting the differences in levels of usage and perceptual abilities (Xu and Huang, 2021; Lee, 2024). The conclusion of this study indicates that improving the network literacy of middle-aged and older adults, especially their skills in using and perceiving the internet, is an effective way to enhance their well-being. Subsequently, the positive and robust impact of network literacy is further verified through the use of instrumental variables and the replacement of the dependent variable. In real life, many middle-aged and older adults have improved their well-being and reduced depression by proficiently using the Internet for information and communication. This further proves the positive impact of network literacy on the psychological state of middle-aged and older adults. In addition, the heterogeneity analysis shows that the positive impact of network literacy on subjective well-being is more significant among middle-aged and older adults who are male, or live in rural areas, or have lower educational attainment or lower income levels. This is likely due to the fact that these groups often face more barriers in accessing and utilizing digital resources. For instance, individuals with lower educational attainment or income levels may have limited opportunities to develop network literacy skills. Similarly, rural residents might suffer from inadequate internet infrastructure (Zhang D. et al., 2022). For these groups, enhancing network literacy can more effectively bridge the digital divide and bring about significant improvements in their subjective well-being by opening up new avenues for information access, social interaction, and economic participation.

Secondly, this study verifies the mediating effect of interpersonal relationships in the impact of network usage literacy and network perception literacy on subjective well-being among middle-aged and older adults. The positive mediating effects account for 26.643 and 31.612%, respectively. It can be seen that the stronger the network literacy ability of middle-aged and older adults, the more helpful it is to promote better interpersonal relationships and thus enhance their subjective well-being. Many previous studies have also shown that using the Internet can reduce loneliness (Schlomann et al., 2020), improve communication with relatives and friends (Becchetti et al., 2008), and obtain more social support (Yang et al., 2023). According to Fowler and Christakis' research, there is a correlation between network relationship centrality and well-being (Fowler and Christakis, 2008), with people at the core of the network being more likely to feel happy, while those at the periphery of the network are more likely to feel unhappy (Yan et al., 2023). Maslow's hierarchy of needs theory states that social interaction needs are one of the fundamental human needs (Gorman, 2010). The Internet has provided a new social platform and method, making it easier for middle-aged and older adults to keep in touch with their family, friends and society and meet their social needs. The improvement of network usage literacy and network perception literacy has promoted social connections, information exchange, social support, emotional communication, convenience of life, self-efficacy, social participation, and identity among middle-aged and older adults, ultimately playing an important role in enhancing their subjective well-being.

Thirdly, this paper further discusses the impacts of the sub-indicators of the two dimensions of network literacy on subjective well-being among middle-aged and older adults. The study finds that both the frequency of Internet use for social interaction and entertainment have significant positive effects on their subjective well-being, that is, the higher the frequency of online social interaction and entertainment among middle-aged and older adults, the stronger their subjective well-being. Meanwhile, the importance of the online social interaction and entertainment in network perception literacy also have significant positive effects on subjective well-being. Social interaction is very important in the daily life of middle-aged and older adults, which has been found in many previous studies (Tiilikainen et al., 2021; Huang et al., 2023). This is also supported by aging theories such as the activity theory and the healthy aging model (Havighurst and Albrecht, 1953; Bryant et al., 2001). Online social interaction such as using WeChat can help middle-aged and older adults keep up with the times and enhance their sense of social participation and self-worth. Online entertainment activities such as watching short videos can enrich their spiritual life and provide pleasant and relaxing experiences. All of these are important sources for promoting the improvement of well-being. However, the study did not find a significant correlation between subjective well-being and online learning, online work, or online business. This does not mean that these activities have no potential value. It might also be due to the fact that their participation in these aspects is too low. Particularly for middle-aged and older adults in rural areas or with lower education or income levels, participation in online learning, work, or business activities is relatively low. This limited engagement makes it challenging to observe significant improvements in their subjective well-being through these specific indicators. With the continuous development of technology, future studies can explore how to optimize these activities in order to enhance the participation and well-being of middle-aged and older adults. For example, providing more user-friendly online learning platforms and job opportunities may attract more middle-aged and older adults to participate, thereby improving their quality of life. At the same time, promoting the improvement of network literacy and enabling them to use the Internet more confidently may indirectly enhance their subjective well-being. Therefore, policy makers and community organizations should attach importance to training in network literacy to help middle-aged and older adults better integrate into the digital society.

Although this study has provided important insights into the relationship between network literacy and subjective well-being among middle-aged and older adults, there are still some shortcomings: Firstly, the measurement of network literacy relies solely on questions from the CFPS, which does not fully reflect the true level of network literacy, and there are certain differences in the questions related to network literacy in 2020 compared to 2014 and 2018. Secondly, the insignificant impacts of online learning, online work, and online business activities on subjective well-being might be due to the limitations of sample selection. The generally low participation of the middle-aged and older adults in these activities may lead to bias in the results. Finally, the data used in this study are merely based on large sample survey data from China and cannot represent the situation in other countries around the world. Therefore, in the future, comprehensive investigation and research on network literacy should be strengthened, and the sample range should be expanded to consider middle-aged and older adults in different economic and social backgrounds in order to assess the potential impact of these activities more comprehensively. Follow-up studies should consider incorporating the multi-dimensional characteristics of social networks into the analysis to understand their impact on the well-being of middle-aged and older adults more comprehensively.

## 5 Conclusion

Based on the data from the China Family Panel Studies (CFPS), this study explores the correlation between network literacy and subjective well-being among middle-aged and older adults, and analyzes the mediating effect of interpersonal relationships. The research has found a significant positive correlation between network literacy and subjective well-being, with both network usage literacy and network perception literacy positively promoting their well-being, particularly among middle-aged and older adults who are male, or live in rural areas, or have lower educational attainment or lower income levels. Further analysis shows that interpersonal relationships play a positive mediating role between network literacy and subjective well-being, indicating that the improvement of network literacy helps middle-aged and older adults establish better social relationships, thereby enhancing their well-being. In addition, the frequency of social and entertainment activities in network usage literacy, as well as the importance of social and entertainment activities in network perception literacy, have particularly significant impacts on subjective well-being. The findings highlight the importance of enhancing network literacy among middle-aged and older adults, offering a foundation for policy makers and community organizations to support their integration into the digital society, enjoy the convenience and fun brought by the Internet, and thus improve their quality of life and well-being. In the context of increasing aging, future research should continue to focus on the multidimensional development of network literacy and its long-term impact on the well-being of middle-aged and older adults.

## Data Availability

Publicly available datasets were analyzed in this study. The data we use is from the China Family Panel Studies (CFPS), which is available at the website: https://opendata.pku.edu.cn/dataverse/CFPS?language=en.
